# Hypoglycemia and the Origin of Hypoxia-Induced Reduction in Human Fetal Growth

**DOI:** 10.1371/journal.pone.0008551

**Published:** 2010-01-01

**Authors:** Stacy Zamudio, Tatiana Torricos, Ewa Fik, Maria Oyala, Lourdes Echalar, Janet Pullockaran, Emily Tutino, Brittney Martin, Sonia Belliappa, Elfride Balanza, Nicholas P. Illsley

**Affiliations:** 1 Department of Preventive Medicine and Community Health, University of Medicine and Dentistry-New Jersey, Newark, New Jersey, United States of America; 2 Department of Obstetrics Gynecology and Women's Health, University of Medicine and Dentistry-New Jersey, Newark, New Jersey, United States of America; 3 Hospital Hernandez Vera, Villa Primer de Mayo, Santa Cruz, Bolivia; 4 Instituto Boliviano de Biología de Altura, Universidad de San Andreas Mayor, La Paz, Bolivia; Sun Yat-Sen University, China

## Abstract

**Background:**

The most well known reproductive consequence of residence at high altitude (HA >2700 m) is reduction in fetal growth. Reduced fetoplacental oxygenation is an underlying cause of pregnancy pathologies, including intrauterine growth restriction and preeclampsia, which are more common at HA. Therefore, altitude is a natural experimental model to study the etiology of pregnancy pathophysiologies. We have shown that the proximate cause of decreased fetal growth is not reduced oxygen availability, delivery, or consumption. We therefore asked whether glucose, the primary substrate for fetal growth, might be decreased and/or whether altered fetoplacental glucose metabolism might account for reduced fetal growth at HA.

**Methods:**

Doppler and ultrasound were used to measure maternal uterine and fetal umbilical blood flows in 69 and 58 residents of 400 vs 3600 m. Arterial and venous blood samples from mother and fetus were collected at elective cesarean delivery and analyzed for glucose, lactate and insulin. Maternal delivery and fetal uptakes for oxygen and glucose were calculated.

**Principal Findings:**

The maternal arterial – venous glucose concentration difference was greater at HA. However, umbilical venous and arterial glucose concentrations were markedly decreased, resulting in lower glucose delivery at 3600 m. Fetal glucose consumption was reduced by >28%, but strongly correlated with glucose delivery, highlighting the relevance of glucose concentration to fetal uptake. At altitude, fetal lactate levels were increased, insulin concentrations decreased, and the expression of GLUT1 glucose transporter protein in the placental basal membrane was reduced.

**Conclusion/Significance:**

Our results support that preferential anaerobic consumption of glucose by the placenta at high altitude spares oxygen for fetal use, but limits glucose availability for fetal growth. Thus reduced fetal growth at high altitude is associated with fetal hypoglycemia, hypoinsulinemia and a trend towards lactacidemia. Our data support that placentally-mediated reduction in glucose transport is an initiating factor for reduced fetal growth under conditions of chronic hypoxemia.

## Introduction

Abnormalities of fetal growth cost billions per year in neonatal intensive care, maternal hospital admissions and lost work productivity for the worried parents. The subsequent financial and social costs due to long-term damage to the fetus, e.g. physical handicaps and/or diminished cognitive abilities, are inestimable [1]. Intrauterine growth restriction (IUGR) affects 3–10% of pregnancies with rates steadily increasing in the USA [2]; 20% of stillborn infants have IUGR [3]. Perinatal mortality rates are 4–8 times higher for growth retarded infants, and morbidity is present in 50% of surviving infants [3], [4], [5], [6]. To date no intervention other than delivery has proved effective. A common underlying contributor to IUGR is placental hypoxia due to impaired placental invasion of maternal blood vessels and/or poor placental vascular development that diminishes the fetal vascular surface area available for oxygen diffusion [7]. Determining the effects of chronic hypoxia on human fetoplacental metabolism and growth has long presented a challenge, due not only to ethical concerns and problems of accessibility, but because hypoxia is almost always present in combination with additional pathologies such as abnormal placental development or preeclampsia. The natural experiment afforded by human residence at high altitude allows us to study fetal and placental responses to chronic hypoxia in the absence of these additional pathologies. This report describes fetal glucose delivery and consumption in low and high altitude pregnancies in which we have carefully documented the fact that there is no fetal oxygen deficit at high altitude and thus that hypoxia is not the proximate cause of the reduction in fetal growth.

Pregnancy at >2700 m altitude is associated with reduced third trimester fetal growth; these babies do not attain their genetic growth potential during intrauterine life [8]. Previous studies in diffusion-dependent avian models demonstrate that it is the high-altitude environment, specifically reduced oxygen tension [9] that ultimately causes the reduction in fetal growth, as growth restriction is reversible with supplemental oxygen. Other models, where the placenta can profoundly modulate gas and nutrient delivery to the fetus, show variable degrees of growth restriction at high altitude depending on the species, timing of exposure etc. In the sheep model, for example, maternal and fetal hematological adaptations defend fetal oxygen supply and permit normalization of fetal growth [10], [11]. This is not the case in humans. Altitude diminishes birth weight by 100 grams per 1000 m gain in elevation [12], and the effect is independent of maternal attributes and socioeconomic characteristics [9], [13].

We have shown that placental and fetal hypoxia are present in human high-altitude pregnancy [14], [15], [16]. However, the reduced growth of an otherwise healthy fetus in the third trimester is not due to a reduced quantity of oxygen delivered to or consumed by the fetus [8], [17]. Although the idea that deficit in maternal oxygen transport is the causal mechanism for reduced fetal growth has been promoted by one group [18], [19], numerous experimental animal studies show that maternal blood flow to the uteroplacental unit can be reduced by up to 50% without reduction in fetal growth [reviewed in 20]. We recently published the first studies of maternal and fetal oxygen delivery and consumption in genetically matched women at low versus high altitude. We found that maternal oxygen delivery to the uteroplacental unit is 5-fold greater than fetal consumption and that fetal oxygen delivery and consumption is the same at low versus high altitude [8], [17]. We found that, as in the sheep model, the human fetus vigorously defends its oxygen supply. Thus despite an approximately 40% reduction in maternal arterial oxygen tension, umbilical venous oxygen tension was reduced by only 10% [10], within one standard deviation of normal sea level values [21]. These data point to alterations in placental function and/or metabolism as both defending fetal oxygenation while at the same time reducing fetal growth.

In view of the reduction in fetal weight at high altitude, the absence of changes in fetal oxygen delivery and consumption raises a crucial question. What is the mechanism for the reduction in fetal growth? After oxygen, glucose is the most important substrate for fetal growth, and the only one that can be metabolized anaerobically. We hypothesized that the reduction in fetal growth in conditions of chronic hypoxia is associated with decreased delivery of glucose to the fetus and decreased fetal glucose consumption. We hypothesized also that the decrease in fetal glucose delivery was a result of increased anaerobic metabolism of glucose by the placenta.

## Materials and Methods

### Ethics Statement

All participants gave written, informed consent to protocols approved by the collaborating Bolivian institution (Instituto Boliviano de Biología de Altura, Consejo Tecnico), the Bolivian National Bioethics Committee and the Institutional Review Board of the New Jersey Medical School.

### Study Design/Participants

The study reported here is part of a larger, cross-sectional, prospective study designed to evaluate the effect of altitude on uterine arterial and umbilical blood flows, oxygen delivery and consumption and on fetal and placental growth in adapted (Native American) versus non-adapted (European Hispanic) populations living at low and high altitude in Bolivia [8], [17]. The blood flow, oxygen delivery and oxygen consumption data have been published for the full sample, and showed that while blood flow and oxygen delivery were greater in Andean women, regardless of altitude, changes attributable to altitude were the same between ancestry groups [8], [17].

The participants included in this report are 69 mother-infant pairs living at 400 m altitude (Santa Cruz, Bolivia) and 58 at 3600 m (La Paz, Bolivia), the subset in whom maternal and fetal arterial and venous plasma samples were successfully collected and measured for glucose, lactate and insulin. In this report we present the relevant data on blood flows, oxygen delivery and consumption for this subset.

Inclusion criteria were singleton pregnancy, good health (absence of obstetric or medical complications including chronic conditions such as hypertension, renal disease, obesity), a normal oral glucose tolerance test, conception, gestation and delivery at the altitude of study and elective cesarean delivery without labor at term. Women were excluded if labor began prior to cesarean delivery, for drug, alcohol or tobacco use, or if they developed complications of pregnancy.

### Study Protocol

Prior to delivery (4±1days, range, 0–10), we obtained a medical/obstetric history, evaluated general measures of health (blood pressure, oxygen saturation, urinalysis) and completed a research ultrasound that measured bilateral maternal uterine blood flow and fetal umbilical venous blood flow in fasting women as previously published [8], [17], [22]. Maternal “arterialized” (warmed hand vein) and venous blood was obtained prior to delivery for measurement of blood gases, glucose, lactate, insulin and for determinations of hematocrit and hemoglobin, required for calculation of oxygen content and delivery. Using ‘arterialized’ maternal venous blood, obtained after warming the hand vein, is a widely practiced technique for obtaining mixed venous and arterial blood that largely reflects arterial blood values, especially for metabolites [23], [24]. It is frequently used where it is impossible, impractical or clinically and/or ethically unacceptable to sample arterial blood directly. It has been used frequently in pregnancy to obtain values for maternal arterial blood gases (see for example [25]). At delivery the doubly-clamped cord and placenta were immediately sampled for blood from the umbilical vein and artery and for tissue samples from the villous core. Samples for blood gas analyses were collected into heparinized blood gas syringes which were sealed and immediately placed on ice. Blood gases were measured within 1 hour of collection. Samples for glucose and lactate were collected into tubes containing calcium oxalate and into lithium heparin for insulin determinations. Plasma for these latter three assays was separated immediately, then frozen at −80°C until assay. The acceptance criteria for the venous and arterial/arterialized samples was that oxygen was not administered to the mother prior to blood collection or clamping of the umbilical cord, that arterial pH differed from venous by >0.02, and that arterial PCO_2_ was ≥3.8 mmHg higher than venous PCO_2_ [21]. This ensured that cord blood samples were not inadvertently collected from the same vessel, or from mixed venous and arterial cord blood. Blood gases were measured in duplicate using a Radiometer ABL 5000 (Copenhagen, Denmark) at high altitude and an Eschweiler ECOSYS II (Kiel, Germany) at low altitude; the machines were calibrated prior to every study using the manufacturers' standard calibration solutions. The CV of duplicate measures did not exceed 2% for any blood gas or hematological parameters. Hemoglobin was measured using a Radiometer OSM 3 (Copenhagen, Denmark) calibrated daily using the manufacturers standards, or by the cyanomethemoglobin technique.

Placental weight was measured after trimming the cord and membranes. Ponderal index, which estimates neonatal body mass, was calculated as 100 * birth weight (g)/birth length (cm)^3^. All subjects in this study were delivered under spinal or epidural anesthesia without supplemental oxygen. Placental tissue samples were obtained and syncytiotrophoblast basal plasma membranes were prepared as described previously [26] for analysis of the GLUT1 glucose transporter, the only functional glucose transporter at the maternal-fetal interface.

The ultrasound methods for measurement of volumetric blood flow have been described in detail in our prior publications [8], [17], [22]. Briefly, we used ultrasound and Doppler to measure the diameter and blood mean flow velocity in the left and right maternal uterine arteries and in the fetal umbilical vein to calculate volumetric blood flow to the pregnant uterus and to the fetus [27], [28]. These techniques have been validated in prior studies using measurements obtained directly by flow probe [29]. Volumetric flow (ml.min^−1^) was calculated as the cross-sectional area of the blood vessel x mean flow velocity x 60. Blood flows and oxygen delivery were summed for the left and right uterine arteries within each participant and are presented as bilateral flow and oxygen delivery.

Plasma glucose concentrations were measured using a hexokinase-glucose-6-phosphate dehydrogenase coupled enzyme assay [30]. Plasma lactate was determined using a lactate dehydrogenase enzymatic assay [31]. Plasma insulin concentration was measured by a commercial ELISA (Diagnostic Systems Laboratories, Webster, TX), according to the manufacturer's instructions. In each assay a pooled sample from all subjects was run in triplicate to measure inter-assay variation. Intra-assay variation in triplicate samples was 3±1% for glucose, 2±1% for lactate and 5±1% for insulin. Inter-assay variation was 4±2% for glucose, 3±1% for lactate and 8±2% for insulin. The mean (or median) differences observed between groups in these parameters were substantially greater than the variations due to assay errors. GLUT1 glucose transporter expression was measured in the syncytial basal membrane by slot immunoblotting using a polyclonal antibody to GLUT1 (Chemicon, Temecula, CA) as described previously [15].

Maternal glucose delivery to the uteroplacental unit was calculated as maternal bilateral uterine blood flow multiplied by arterial glucose concentration and normalized to uterine contents (birth weight plus placental weight) in units of mmol.min^−1^.kg^−1^. Fetal glucose delivery was calculated as umbilical venous blood flow multiplied by umbilical venous concentration of glucose (mmol.min^−1^). Fetal glucose consumption was calculated as the umbilical venous-arterial difference in glucose concentration multiplied by umbilical blood flow and normalized to birth weight (µmol.min^−1^.kg^−1^).

### Statistics

Because the parent project involved comparisons of blood flow and oxygen delivery between four groups, Andean Native Americans versus Hispanics of European ancestry living at high versus low altitude [8], [17], our initial statistical analyses were conducted to ascertain whether or not any of the dependent variables in this study of glucose transport and consumption differed according to ancestry. For only one of the dependent variables, umbilical arterial lactate, was there interaction between altitude and ancestry; the altitude-associated increase in lactate was greater in the Hispanic than Andean women. Therefore the ancestry groups were combined to increase statistical power and the data reported here are based on altitude differences only. Our prior studies showed that while blood flow and oxygen delivery were greater in Andean women, regardless of altitude, changes attributable to altitude were the same between ancestry groups. Although the ancestry groups were combined to obtain the data reported in this study, the ancestry-specific data at each altitude is reported in supplementary data tables for the interested reader (Tables S1-S3).

At each altitude, most of the continuous data presented in this report were sampled from a population which approximated a Gaussian distribution as indicated by the p value derived from the Kolmogorov-Smirnoff statistic (p>0.1) except for GLUT1 glucose transporter expression and insulin concentrations. Within each group maternal age, height, pre-pregnant BMI, weight gain with pregnancy, parity, infant sex, and gestational age at birth were analyzed using multiple regression to evaluate the impact of maternal and fetal characteristics on birth weight. Birth weights were then adjusted for significant contributors to fetal weight (maternal age, parity, infant sex and gestational age). The difference in the male/female distribution between altitudes was analyzed by Fishers Exact test. Glucose and lactate concentrations were compared between altitudes using a two-tailed, unpaired Student's *t* test. The Mann-Whitney U test was used to compare GLUT1 glucose transporter expression and the plasma insulin values. Because the initial statistical analyses compared 4 groups, a Bonferroni correction was applied to the altitude comparisons reported here and the p value accepted for significance was <0.01. Correlation analyses were used to evaluate relationships between independent and dependent continuous variables. Normally distributed data in figures and tables are presented as mean ± SEM; insulin and glucose transporter and insulin data are presented as medians and inter-quartile range.

## Results

### Ethnicity and Altitude

Of the demographic and neonatal characteristics considered in relation to birth weight, maternal age, BMI, height, weight gain, parity and infant sex explained 26% of the variation in birth weight among Europeans at high altitude (p = 0.15), 18% in Andean at low altitude (p = 0.24) and 24% in Andean at high altitude (p = 0.15). Taller European women at high altitude had larger babies (r^2^ = 0.12, p<0.05) and males were heavier than females (r^2^ = 0.07, p = 0.11), regardless of altitude. Greater BMI in Andean women at low altitude contributed to greater birth weight (r^2^ = 0.12, p<0.05) and greater parity among Andean women at high altitude was associated with greater birth weight (r^2^ = 0.12 p<.05). We therefore adjusted the individual birth weights within each group, controlling for these variables and for gestational age (Table S1). Controlling for differences in maternal and neonatal characteristics between groups, the altitude-associated decrement in birth weight was −442 grams for Europeans and −237 grams for Andeans.

Apart from neonatal anthropometry, only one of the dependent variables presented in this paper, umbilical arterial lactate, showed interaction between altitude and ancestry; the altitude-associated increase in lactate was greater in the European than Andean women. Therefore the ancestry groups were combined to increase statistical power and the data reported here reflect altitude differences only. The ancestry-specific data at each altitude is reported in supplementary data tables for the interested reader (Table S2, S3).

### Maternal and Neonatal Characteristics

High altitude mothers were older (4±1 yrs; p<0.0001) and shorter (4±1 cm; p = 0.02) than their low-altitude counterparts, but comparable in all other respects (Table 1). The altitude-associated decrement in birth weight was 326 grams. The growth restriction was asymmetric as birth length and body weight were reduced but head circumference was similar at 400 versus 3600 m. Placental weight was similar, but the birth to placental weight ratio was reduced, thus the placenta was larger for any given fetal weight at 3600 m (Table 1). The unequal distribution of sexes between the low and high altitude populations should be noted, however it is not statistically significant. Table 1 indicates that the adjusted birth weights between altitudes, corrected for this uneven distribution, were only minimally different from the raw birth weights.

**Table 1:** Maternal and infant characteristics.

Maternal characteristics	400 m	3600 m	p values
	n = 69	n = 58	
Gestational age at ultrasound	38.0±0.2	38.0±0.2	= 0.84
Age (years)	28±1	32±1 *	<0.0001
Parity (#), (% primiparous)	1.2±0.2 (39%)	1.2±0.10 (32%)	= 0.87, 0.78
Height (cm)	159±1	155±1	= 0.02
Non-pregnant weight (kg)	61±1	59±1	= 0.50
Non-pregnant Body Mass Index (kg.m^−2^)	24±1	25±1	= 0.51
Weight gain with pregnancy (kg)	12±1	13±1	= 0.38
Infant characteristics			
Birth weight (grams, (unadjusted values)	3479±49	3180±53 *	<0.0001
Birth weight (grams, adjusted values)	3485±23	3159±38 *	<0.0001
Placental weight (g)	465±11	498±15	= 0.07
Birth/placental weight ratio	7.7±0.2	6.6±0.2 *	<0.0001
Ponderal index	2.69±0.03	2.78±0.04	= 0.09
Birth length (cm)	50.5±0.2	48.6±0.2 *	<0.001
Abdominal circumference (cm)	34.1±0.2	33.7±0.2	= 0.21
Head circumference	34.7±0.2	34. 5±0.2	= 0.38
Clinically assessed gestational age at birth (wks)	38.6±0.1	38.6±0.1	= 0.62
Days (from LMP)	272±1	271±1	= 0.36
Sex ratio M/F	34/35	24/34	= 0.37

### Blood Flows

Pregnant women at high altitude had reduced bilateral uterine artery volumetric blood flow (Table 2). However, increases in maternal hemoglobin concentration raised maternal arterial oxygen content at 3600 m (6.8±0.1 vs. 7.9±0.1 mmol.L^−1^ at 400 m and 3600 m, respectively, p<0.001) and this compensated for reduced blood flow so that uteroplacental O_2_ delivery was similar between altitudes (Table 2). Fetal umbilical venous blood flow was also reduced at high altitude. However, as in the mothers, an increase in fetal oxygen content (4.5±0.1 at 400 m vs. 5.5±0.2 mmol.L^−1^ at 3600 m, p<0.01), among other adaptations previously reported [17], preserved oxygen delivery and consumption to comparable values between altitudes (Table 2).

**Table 2:** Blood flows, oxygen delivery and consumption.

	400 m	3600 m	p values
	n = 69	n = 58	
Bilateral maternal uterine blood flow (ml.min^−1^.kg^−1^ uterine contents [fetal + placental weight])	172±9	136±8	<0.005
Uteroplacental O_2_ delivery (mmol.min^−1^.kg^−1^ uterine contents)	1.2±0.1	1.1±0.1	= 0.36
Fetal blood flow (ml.min^−1^.kg^−1^ fetal weight)	100±4	80±3	<0.0001
Fetal oxygen delivery (mmol.min^−1^.kg^−1^ fetal weight)	0.44±0.02	0.44±0.02	= 0.93
Fetal oxygen consumption (mmol.min^−1^.kg^−1^ fetal weight)	0.27±0.01	0.27±0.01	= 0.97

### Maternal Glucose

**Figure 1:**
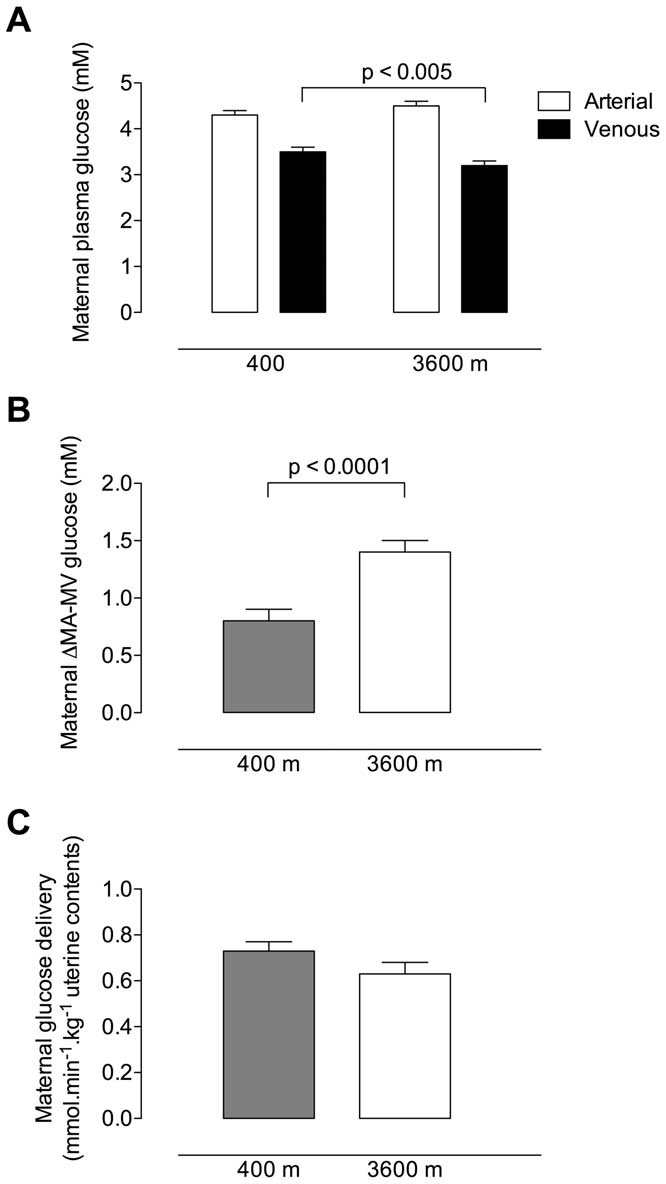
Maternal glucose concentrations and uterine glucose delivery. (A) Maternal arterial and venous plasma glucose concentrations in normal, term pregnancies at low vs. high altitude. Arterial glucose concentrations were similar at low (4.3±0.1 mM) and high altitude (4.5±0.1 mM, p = 0.06), but venous concentrations were reduced at high altitude (3.5±0.1 at 400 m and 3.2±0.1 at 3600 m, p<0.005). (B) Maternal arterial-to-venous differences in plasma glucose concentration were lower at 400 m (0.85±0.1 mM) than at 3600 m (1.40±0.1 mM, p<0.0001). (C) Maternal uterine glucose delivery was similar at 400 m (0.73±0.04 mmol.min^−1^.kg^−1^ uterine contents) and 3600 m (0.63±0.04 mmol.min^−1^.kg^−1^ uterine contents, p = 0.06).

Maternal arterial glucose concentrations were similar at low versus high altitude (Fig. 1A). Maternal venous glucose concentrations were significantly lower at 3600 m than at 400 m. The arterial to venous (MA-MV) difference in glucose was therefore greater at high altitude (Fig. 1B), suggesting that the high altitude mothers were extracting more glucose from the arterial to venous circulation than the low altitude mothers. Despite the reduction in uterine blood flow (Table 2), maternal glucose delivery to the uteroplacental circulation was similar at low versus high altitude (Fig. 1C). Maternal arterial and venous glucose concentrations were positively correlated (r^2^ = 0.36, p<0.001, low altitude; r^2^ = 0.24, p<0.005, high altitude).

### Fetal Glucose

**Figure 2:**
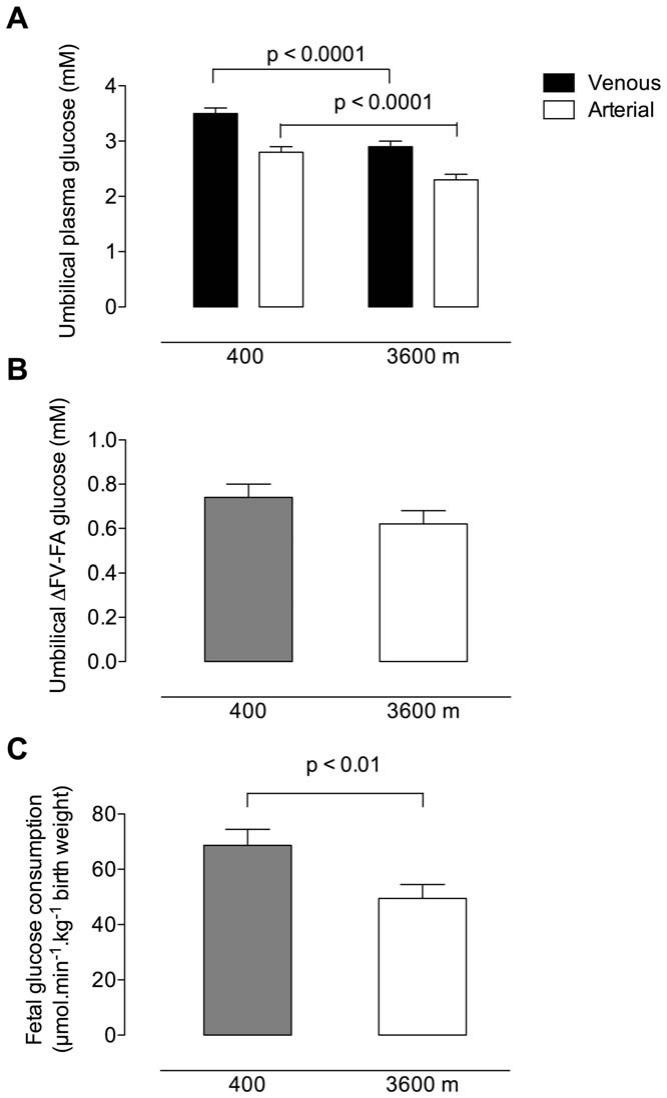
Fetal glucose concentrations and fetal glucose consumption. (A) Umbilical venous and arterial plasma glucose concentrations in normal, term pregnancies at 400 and 3600 m. Glucose concentrations were greater in the umbilical vein at 400 m (3.5±0.1 mM) than at 3600 m (2.9±0.1 mM, p<0.0001). Fetal umbilical arterial glucose concentrations were also greater at low (2.8±0.1 mM) than at high altitude (2.8±0.1 vs. 2.3±0.1 mM, p<0.0001). (B) Fetal venous-to-arterial differences in plasma glucose concentration were similar at 400 m (0.74±0.06 mM) and 3600 m (0.62±0.06 mM), p = 0.16). (C) Fetal glucose consumption at was greater at 400 m than 3600 m.

Glucose concentrations in both the umbilical vein and artery were decreased at high altitude (Fig. 2A). The magnitude of the decrease between the umbilical venous and arterial circulations was similar, thus the fetal venous to arterial difference (FV-FA) in glucose concentration did not differ between altitudes (Fig. 2B). Also notable is the fact that the maternal arterial-fetal arterial (MA-FA) glucose gradient at high altitude is much greater than that observed at low altitude (2.11±0. 15 mM vs. 1.51±0.10 mM, p<0.001). Umbilical venous and arterial glucose concentrations were positively correlated (r^2^ = 0.65, p<0.001, low altitude; r^2^ = 0.43, p<0.001, high altitude). Glucose extraction by the fetus is equivalent to glucose consumption, as there is no gluconeogenesis in the human fetus [32], [33], thus with knowledge of fetal volumetric blood flows we can calculate fetal glucose consumption. Using the FV-FA differences in glucose concentration and the associated fetal blood flows (Table 2), we found that chronic hypoxia caused a decrease in fetal glucose consumption from 68.7±5.8 to 49.5±6.6 µmol.min^−1^.kg^−1^ (Fig. 2C; p<0.01).

**Figure 3:**
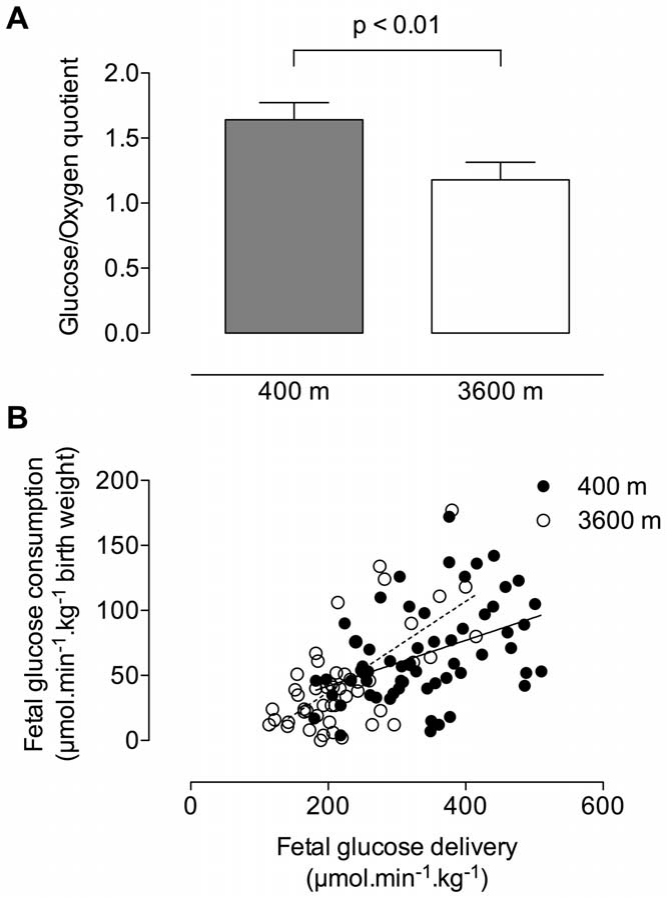
Indicators of fetal glucose metabolism. (A) The fetal glucose/oxygen quotient (6 x fetal glucose consumption/fetal oxygen consumption) was greater at 400 m (1.64±0.13) than at 3600 m (1.18±0.13 p<0.01). (B) Fetal glucose delivery and fetal glucose consumption were positively correlated both altitudes (y = 0.08+0.176x, r^2^ = 0.18, p<0.001 at 400 m; y = 0.23+0.35x, r^2^ = 0.43, p<0.0001 at 3600 m).

Neither birth nor placental weights were associated with maternal or fetal glucose concentrations, nor with the consumption of glucose at either altitude. There was no relationship between oxygen and glucose consumption at either altitude. When we examined glucose consumption relative to oxygen consumption (glucose:oxygen quotient) we found that the high altitude fetus was consuming less glucose per mole of oxygen at high versus low altitude (Fig. 3A). Fetal glucose delivery was positively associated with fetal glucose consumption at both altitudes but the slope of the relationship was 2-fold greater in high compared to low altitude pregnancies (Fig. 3B).

### Metabolic Responses

**Figure 4:**
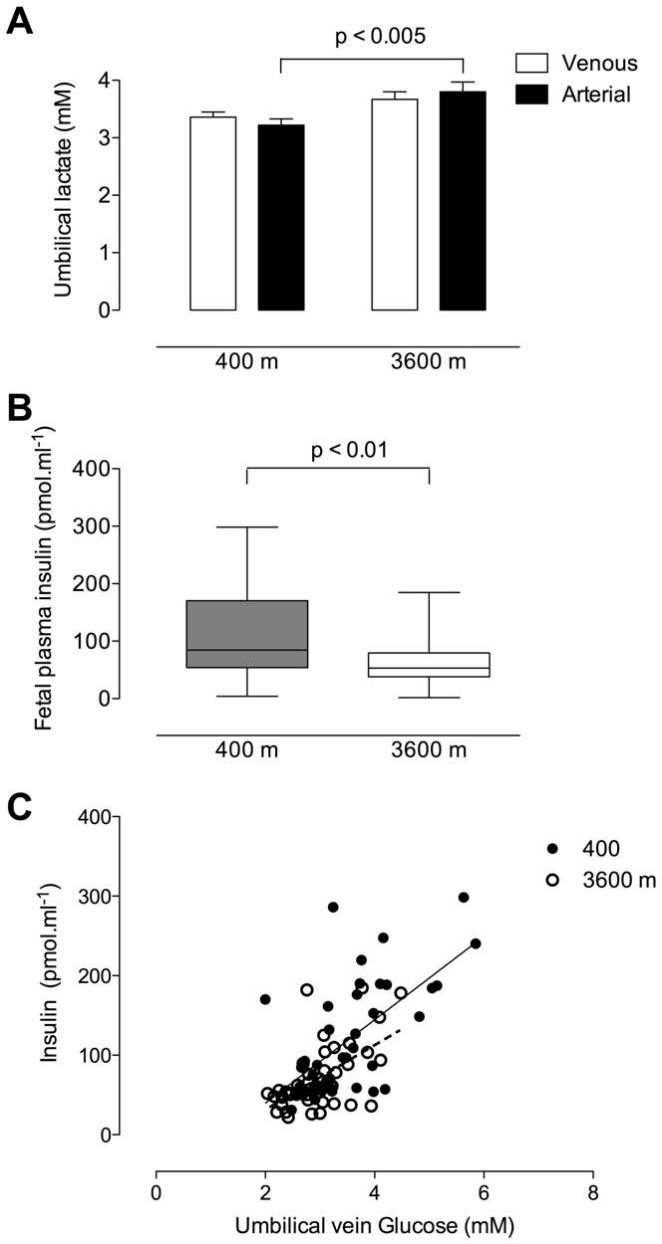
Fetal lactate and insulin response to altered glucose delivery and consumption. (A) Umbilical venous plasma lactate concentrations in normal, term pregnancies at 400 vs 3600 m did not differ (3.36±0.09 mM at 400 m and 3.67±0.13 mM at 3600 m, p = 0.06), whilst arterial concentrations were lower at 400 m (3.22±0.11 mM) than at 3600 m (3.80±0.17, p<0.005). (B) Insulin concentrations were greater in the low vs. high altitude fetus (low altitude, median  = 84.7 pmol.ml^−1^ [54.1–170.1]; high altitude, median  = 53.5 pmol.ml^−1^ [38.2–79.2]). (C) Fetal glucose and insulin concentrations were positively correlated at both altitudes (400 m y  = −65.2+52.4x r^2^ = 0.34, p<0.0001; 3600 m y  = −49.4+40.5x, r^2^ = 0.33, p<0.0001) but neither the slopes (p = 0.42) nor intercept (p = 0.11) differed.

To determine whether these changes in fetal glucose concentrations/consumption affected fetal metabolic response, we measured the lactate concentrations in the umbilical vein and artery (Fig. 4A). Umbilical venous lactate was similar between altitudes, whilst umbilical arterial lactate concentration was increased at high altitude. As a result of the wide range of plasma lactate values, net lactate fluxes were not significantly different from zero at either low or high altitude. Umbilical arterial lactate concentration was negatively correlated with umbilical arterial O_2_ content, but the slopes and intercepts were similar between altitudes (y = 4.5 − 1.2(x)+0.2(x^2^), r^2^ = 0.24, p<0.005 at 400 m; y = 4.9 − 1.0(x)+0.15(x^2^), r^2^ = 0.20, p<0.005) at 3600 m).

As a means of assessing the fetal endocrine response to the changes in glucose delivery and consumption, we measured umbilical venous plasma insulin concentrations. Insulin concentrations were decreased in high versus low altitude neonates (Fig. 4B). Umbilical venous glucose and insulin concentrations were positively correlated (Fig. 4C), but neither the slopes nor intercept differed at low vs. high altitude, suggesting that fetal insulin response to glucose was similar at low and high altitude.

### Glucose Transporters

Previously, in a smaller sample with a reduced altitude difference (1600 vs 3100 m), we showed that expression of the GLUT1 glucose transporter on the basal membrane of the syncytiotrophoblast was reduced by ∼40% [15]. We repeated these measurements by immunoblotting of the isolated placental syncytiotrophoblast basal membranes from a randomly chosen subset of the pregnancies described in Table 1. Expression of GLUT1 decreased from a median value of 1.91 [1.03–6.03] at low altitude, to 1.03 [0.68–2.14] at high altitude (arbitrary units; p<0.01; n = 30, 32).

## Discussion

The birth weight of neonates at 3600 m is reduced >300 g compared to low altitude pregnancies, despite the fact that maternal and fetal oxygen delivery and fetal oxygen consumption do not differ between altitudes. Maternal glucose delivery to the placenta did not differ between altitudes but both umbilical venous and arterial glucose concentrations were significantly decreased at high altitude. This was associated with a >28% reduction in fetal glucose consumption, a 37% decrease in median fetal plasma insulin concentrations and a trend towards fetal lactacidemia at high altitude. The hypothesis that reduced fetal weight was associated with decreased fetal glucose delivery and consumption was supported. The hypothesis that increased metabolism of glucose by the placenta contributes to the reduction in glucose consumption was supported by the observation of decreased delivery of glucose to the high altitude fetus despite a greater maternal arterial to fetal arterial gradient. Finally, the hypothesis that there might be increased anaerobic metabolism of glucose by the placenta at high altitude was supported by decreased delivery of glucose in the absence of changes in oxygen delivery to placenta and fetus. The amount of glucose delivered to the fetus was strongly associated with the amount of glucose consumed, presumably contributing to the growth (or lack thereof) of the fetus. The relationship between glucose delivery and consumption was far more pronounced at high altitude, suggesting that glucose dependency in the high altitude fetus may be increased relative to the low-altitude fetus. In contrast, the amount of insulin secreted relative to fetal glucose concentrations did not differ between altitudes, indicating that decreased fetal growth is not attributable to alterations in the fetal insulin response. These results lead us to conclude that the reduction in fetal growth due to chronic hypoxia is mediated not by limitations on oxygen availability, but by fetal hypoglycemia and its consequences. The model of high altitude pregnancy reveals that an initiating step in IUGR may be decreased glucose delivery to the fetus, presumably as a result of the increased placental consumption of the glucose delivered by the mother to the uteroplacental circulation.

As is often the case in human studies our ability to definitively prove the hypotheses above is limited by ethical and access concerns. Ideally, samples from the uterine vein would have permitted a direct calculation of total uteroplacental glucose and oxygen uptake allowing a direct partition between uteroplacental and fetal glucose and oxygen uptake. Such samples are rarely acquired in humans, even in the most sophisticated of medical settings and were not ethically permissible nor feasible under the conditions extant in Bolivia. Likewise blood flow measurements are known to be variable. We took great care to measure our pregnant women as close to elective cesarean delivery as possible, in the same position and at the same time of day; replicate measures and our coefficients of variation were similar to those previously published [34], [35], [36]. The un-normalized bilateral uterine blood flow measurement reported here of 670±32 vs. 505±30 ml.min^−1^ at low and high altitude respectively are entirely consistent with the several prior studies using multiple methodologies that converge on a mean value of ∼700 ml.min^−1^ (reviewed in [37]). It is reassuring that the fetal blood flow measurements reported in Table 2 fell, on average, at the 75^th^ percentile (400 m) and the 60^th^ percentile (3600 m) relative to a large, population based analysis of serially studied pregnancies [34] and that our oxygen consumption measurements were virtually identically to the only other human data, recently published [38].

Our data showed that maternal arterial glucose concentrations were marginally greater, but venous concentrations were markedly reduced at high altitude, suggesting greater MA-MV extraction of glucose at 3600 m. We have no measures in the non-pregnant state so this could be a generalized attribute of high altitude residence. However, the only other report on plasma glucose showed that the MA-MV difference in the non-pregnant condition was equivalent at sea level and 4300 m altitude, but increased progressively with gestational age [39]. This supports the possibility that increased placental extraction and consumption of glucose accounts for the increased MA-MV difference. In a prior study, maternal plasma insulin was lower in high compared with low altitude pregnancy [40], indicating that the reduced maternal venous glucose concentration is not a function of an insulin-stimulated increase in glucose uptake in pregnancy. This supports that the cause of the increased MA-MV difference in glucose concentration during pregnancy at high altitude is increased glucose extraction by the uteroplacental circulation.

The umbilical FV-FA difference in glucose concentration we report here is similar to those obtained in prior studies [32], [41], [42], [43], [44]. Previous studies on the association between fetal glycemic status and fetal growth have clearly shown that reduced fetal blood glucose concentrations are associated with fetal growth restriction [44], [45], [46], [47], but these measurements were obtained after the clinical diagnosis of IUGR was made. In our model this difference in fetal metabolic state is present at the time that growth is slowed, but prior to reaching a pathological threshold. In addition, measurement of the corresponding umbilical flows have allowed us to determine, for the first time in humans, fetal glucose consumption. This is also the first direct demonstration that reduced fetal growth is associated with decreased fetal glucose consumption. We show a strong relationship between fetal glucose delivery and consumption, such that greater glucose delivery increases fetal glucose consumption. This causal pathway, coupling fetal hypoglycemia to fetal growth restriction is supported in animal models [48], [49], [50], [51] and there is indirect evidence to demonstrate this relationship in human studies [52], [53], [54]. Complementary to the glucose data is the altitude-associated decrease in fetal plasma insulin, a factor noted consistently in conditions of fetal growth restriction [46], [47], [55]. We have shown that greater fetal glucose concentrations are associated with greater fetal insulin secretion, again highlighting the importance of glucose in moderating fetal growth at any altitude. The conditions of maintained placental weight, fetal oxygenation and oxygen consumption point to the altitude-associated reduction in fetal growth as being initiated by relative hypoglycemia and hypoinsulinemia. It is worth noting that while our 3600 m umbilical venous plasma glucose samples fell at ∼20^th^ percentile for gestational age [44], none were below the threshold for clinical neonatal hypoglycemia, defined as the 5^th^ centile value (1.55 mM) for plasma glucose in neonates 1–2 hr after birth [56].

Despite the increased maternal extraction of glucose at high altitude and similar rates of glucose delivery to the placenta between altitudes, glucose transfer across the placenta at high altitude was only 60% of that measured at low altitude. This implicates the placenta as the source of alterations that lead to the reduced maternal-fetal transfer of glucose. One possible source of the reduced glucose transfer is the decrease in syncytial basal membrane glucose transporter expression, since previous data has suggested that transport across the basal membrane is the rate-limiting step in transplacental glucose transport [57]. We believe that the reduction in basal membrane glucose transporter expression is a secondary effect and not causally related to decreased maternal-fetal glucose transfer, since the effect of hypoxia on trophoblast cells is to increase, not decrease, glucose transporter expression [58], [59].

There are two other possible causes for decreased transfer of glucose into the fetal circulation. The first is decreased delivery to the placenta by the maternal circulation as a result of decreased uterine blood flow and the second is increased placental metabolism of glucose which reduces the level of glucose available for transfer to the fetal circulation. With respect to the former, our previous *in vitro* studies have shown that a decrease in intervillous blood flow consistent with the magnitude of reduction in uterine arterial blood flow at high altitude would only reduce fetal glucose transfer by ∼12% [60]. This would be an overestimation as intervillous blood flow is only a fraction of uterine blood flow. Moreover it should be noted that the maternal arterial-fetal arterial glucose gradient we report at high altitude is much greater than that at low altitude (2.11 vs. 1.51 mM). Since this is a significant factor promoting maternal-fetal transfer [61], [62] and it runs counter to the small decrease likely to result from reduced uterine delivery, it is implausible that the decrease in uterine delivery is responsible for the markedly decreased concentrations in the fetus. The alternative is an increased placental metabolism of glucose, reducing the amount of glucose available for transfer to the fetal circulation. Contrary to previous findings comparing the glucose/oxygen quotient in normal and growth-restricted pregnancies [41], the increased maternal arterial-fetal arterial glucose concentration difference at high altitude is associated with a decreased glucose/oxygen quotient. This means that despite conditions which should promote increased placental transfer, fetal glucose delivery and consumption are decreased, pointing to increased glucose consumption by the placenta as the cause of decreased transport to the fetus. Support for this concept can be drawn from the increased umbilical arterial lactate concentration seen at high altitude. While the net lactate flux data ostensibly demonstrates a switch from fetal consumption at low altitude to fetal production at high altitude, the wide range of plasma lactate values means that neither flux is significantly different from zero. Interestingly however, the inverse correlation between umbilical arterial lactate and O_2_ content reflects the same relationship observed by Marconi et al in IUGR pregnancies [63].

The large change in glucose delivery to the fetus is in marked contrast to fetal oxygen delivery, which is not different between altitudes despite a substantial decrease in maternal arterial oxygen tension from ∼12 kPa to 7 kPa [8], [17]. We suspect that the increased consumption of glucose is accompanied by decreased placental consumption of oxygen, which would explain the relative preservation of a normal fetal oxygen tension and fetal oxygen consumption at 3600 m. These observations are reminiscent of a mechanism known as metabolic reprogramming, in which hypoxia-driven, active inhibition of oxidative metabolism is combined with increased anaerobic use of glucose [64], [65] to produce a hypometabolic state that is reversed when oxygen tension rises. In the models thus far described this mechanism results in increased cellular oxygen availability, enhancing the survival of tumor cells and sparing oxygen to permit increased growth [64] or inducing hypoxia-tolerance in muscle [66], [67]. In the placenta metabolic reprogramming is likely to play a unique and important role in the regulation of fetal growth. The maintenance of fetal oxygenation and the decreased placental transfer of glucose at high altitude are consistent with the operation of such a mechanism in the placenta. Increased availability of oxygen and decreased availability of glucose occur as a result of alterations in placental metabolism, producing a change in the substrate mix provided to the fetus. While increased oxygen availability enhances fetal survival, decreased glucose availability leads to a reduction in fetal growth.

The mechanism described above may be a crucial first step in the processes collectively known as the developmental origins of health and disease [68], whereby an abnormal uterine condition such as hypoxemia results in (semi) permanent changes in adult metabolic and cardiovascular function. In view of the decreased fetal plasma insulin levels observed in the high altitude fetus, it is worth noting the decrease in pancreatic β cell mass and function which accompany hypoglycemia-associated fetal growth restriction in fetal sheep [69], [70], [71]. If β cell dysfunction is associated with hypoglycemia/hypoinsulinemia-mediated reductions in human fetal growth as suggested by these observations, the effect of placentally-mediated alterations in the substrate mix delivered to the fetus s may be carried through to adulthood with the attendant consequences for adult health.

In conclusion, we have shown that chronic (altitude-induced) hypoxia results in decreased fetal circulating glucose concentrations and reduced fetal glucose consumption. Occurring under conditions where oxygenation is sufficient to maintain fetal oxidative metabolism, these data support that hypoglycemia-mediated fetal growth restriction is a result of alterations in placental metabolism.

## Supporting Information

Table S1 This table contains supplementary data on maternal and infant characteristics split by altitude and ancestry.
(0.06 MB DOC)

Table S2 This file contains supplementary data on maternal blood flows, O2 and glucose delivery split by altitude and ancestry.
(0.05 MB DOC)

Table S3 This file contains supplementary data on fetal blood flows, oxygen and glucose delivery split by altitude and ancestry.
(0.05 MB DOC)
