# Supplementary material for: Hypoglycemia and the Origin of Hypoxia-Induced Reduction in Human Fetal Growth
Source: PLoS One. 2010 Jan 1;5(1):e8551. doi: 10.1371/journal.pone.0008551 (PMC2797307; doi:10.1371/journal.pone.0008551)
Supplement: Table S1 — This table contains supplementary data on maternal and infant characteristics split by altitude and ancestry. (0.06 MB DOC) [file pone.0008551.s001.doc]

**Table S1: Maternal and infant characteristics by altitude (alt) and ancestry (anc).**

| Maternal characteristics | 400 m  European n=36 | 3600 m  European  n=27 | 400 m Andean n=33 | 3600 m Andean  n=31 | p values |
| --- | --- | --- | --- | --- | --- |
| Gestational age at ultrasound | 37.7 ± 03 | 37.8 ± 0.3 | 38.4 ± 0.2 | 38.0 ± 0.3 | NS |
| Age (years) | 27 ± 1 | 30 ± 1 | 28 ± 1 | 33 ± 1 | < 0.0001 alt |
| Parity | 0.9 ± 0.2 | 0.9 ± 0.2 | 1.6 ± 0.3 | 1.4 ± 0.3 | < 0.05 anc |
| Height (cm) | 162 ± 1 | 160 ± 2 | 155 ± 1 | 152 ± 1 | < 0.0001 anc |
| Non-pregnant weight (kg) | 62 ± 2 | 61 ± 1 | 59 ± 2 | 58 ± 2 | NS |
| Non-pregnant Body Mass Index (kg.m^-2^) | 24 ± 1 | 24 ± 1 | 25 ± 1 | 25 ± 1 | NS |
| Weight gain with pregnancy (kg) | 12 ± 1 | 12 ± 2 | 12 ± 1 | 13 ± 1 | NS |
| Infant characteristics |  |  |  |  |  |
| Birth weight (grams, (unadjusted values) | 3472 ± 71 | 3001 ± 62 | 3532 ± 51 | 3372 ± 80 | < 0.0001 alt, < 0.01 anc  <.05 interaction |
| Birth weight (grams, adjusted values) | 3412 ± 34 | 2968 ± 39 | 3567 ± 24 | 3345 ± 47 | < 0.0001 alt, < 0.01 anc  <.005 interaction |
| Placental weight (g) | 479 ± 16 | 497 ± 20 | 456 ± 14 | 517 ± 22 | NS |
| Birth/placental weight ratio | 7.4 ± 0.2 | 6.3 ± 0.3 | 7.9 ± 0.2 | 6.8 ± 0.2 | < 0.0001 alt  < 0.05 anc |
| Ponderal index | 2.74 ± 0.04 | 2.60 ± 0.04 | 2.67 ± 0.03 | 2.94 ± 0.06 | < 0.05 anc  < 0.0001 inter |
| Birth length (cm) | 50.2 ± 0.3 | 48.7 ± 0.3 | 51.0 ± 0.2 | 48.6 ± 0.3 | < 0.0001 alt |
| Abdominal circumference (cm) | 34.0 ± 0.2 | 32.8 ± 0.3 | 34.0 ± 0.3 | 34.7 ± 0.2 | < 0.001 inter |
| Head circumference (cm) | 34.7 ± 0.2 | 34.3 ± 0.3 | 34.9 ± 0.3 | 34.8 ± 0.2 | NS |
| Clinically assessed gestational age at birth (wks) | 38.5 ± 0.2 | 38.3 ± 0.2 | 38.8 ± 0.1 | 38.8 ± 0.2 | < 0.05 anc |
| Days (from LMP) | 271 ± 1 | 269 ± 1 | 273 ± 1 | 273 ± 1 | < 0.05 anc |
| Sex ratio M/F | 17/19 | 9/18 | 17/16 | 15/16 | NS |
