# Supplementary material for: Hypoglycemia and the Origin of Hypoxia-Induced Reduction in Human Fetal Growth
Source: PLoS One. 2010 Jan 1;5(1):e8551. doi: 10.1371/journal.pone.0008551 (PMC2797307; doi:10.1371/journal.pone.0008551)
Supplement: Table S2 — This file contains supplementary data on maternal blood flows, O2 and glucose delivery split by altitude and ancestry. (0.05 MB DOC) [file pone.0008551.s002.doc]

**Table S2: Maternal blood flows, O_2_ and glucose delivery by altitude (alt) and ancestry (anc)**

|  | 400 m  European n=36 | 3600 m  European  n=27 | 400 m Andean n=33 | 3600 m Andean  n=31 | p values |
| --- | --- | --- | --- | --- | --- |
| Maternal bilateral uterine artery blood flow (ml.min^-1^) | 621 ± 46 | 456 ± 36 | 723 ± 44 | 551 ± 46 | < 0.001 alt  < 0.001 anc |
| Maternal bilateral uterine artery blood flow (ml.min^-1^.kg^-1^ uterine contents) [placental+fetal weight]) | 160 ± 12 | 129 ± 9 | 186 ± 12 | 142 ± 12 | < 0.005 alt  < 0.09 anc |
| Maternal uteroplacental O_2_ delivery (ml.min^-1^) | 99 ± 8 | 85 ± 7 | 107 ± 7 | 97 ± 7 | NS |
| Maternal uteroplacental O_2_ delivery  (ml.min^-1^.kg^-1^ uterine contents) | 25 ± 2 | 24 ± 2 | 27 ± 2 | 25 ± 2 | NS |
| Maternal arterialized glucose concentration (mM) | 4.3 ± 0.1 | 4.7 ± 0.2 | 4.3 ± 0.1 | 4.4 ± 0.2 | < 0.09 alt |
| Maternal venous glucose concentration (mM) | 3.4 ± 0.1 | 3.5 ± 0.1 | 3.5 ± 0.1 | 3.1 ± 0.1 | < 0.05 inter |
| Maternal arterio-venous Δ glucose (mM) | 0.9 ± 0.1 | 1.1 ± 0.2 | 0.9 ± 0.1 | 1.3 ± 0.2 | < 0.005 alt |
| Maternal uteroplacental glucose delivery (mmol.min^-1^.kg^-1^ uterine contents) | 0.67 ± 0.05 | 0.61 ± 0.05 | 0.80 ± 0.05 | 0.64 ± 0.06 | < 0.05 alt |
