# Supplementary material for: Hypoglycemia and the Origin of Hypoxia-Induced Reduction in Human Fetal Growth
Source: PLoS One. 2010 Jan 1;5(1):e8551. doi: 10.1371/journal.pone.0008551 (PMC2797307; doi:10.1371/journal.pone.0008551)
Supplement: Table S3 — This file contains supplementary data on fetal blood flows, oxygen and glucose delivery split by altitude and ancestry. (0.05 MB DOC) [file pone.0008551.s003.doc]

**Table S3: Fetal blood flows, O_2_ and glucose delivery and consumption and lactate by altitude (alt) and ancestry (anc)**

|  | 400 m  European n=36 | 3600 m  European  n=27 | 400 m Andean n=33 | 3600 m Andean  n=31 | p values |
| --- | --- | --- | --- | --- | --- |
| Umbilical vein blood flow (ml.min^-1^) | 321 ± 17 | 257 ± 20 | 382 ± 18 | 262 ± 12 | <0.0001 alt  = 0.06 anc |
| Umbilical vein blood flow (ml.min^-1^.kg^-1^ fetal weight)) | 93 ± 5 | 83 ± 6 | 108 ± 5 | 79 ± 4 | <0.0001 alt, <0.05 inter |
| Umbilical vein O_2_ delivery (ml.min^-1^) | 31.5 ± 1.9 | 28.7 ± 3.1 | 37.8 ± 1.8 | 35.0 ± 2.8 | <0.01 anc |
| Umbilical vein O_2_ delivery (ml.min^-1^.kg^-1^ fetal weight) | 9.2 ± 0.5 | 9.6 ± 1.0 | 10.7 ± 0.4 | 10.4 ± 0.8 | <0.01 anc |
| Fetal O2 consumption (ml.min^-1^.kg^-1^) | 5.8 ± 0.3 | 6.4 ± 0.5 | 6.2 ± 0.2 | 5.8 ± 0.4 | NS |
| Umbilical vein glucose concentration (mM) | 3.6 ± 0.1 | 3.0 ± 0.1 | 3.4 ± 0.1 | 2.8 ± 0.1 | <0.0001 alt |
| Umbilical artery glucose concentration (mM) | 2.8 ± 0.1 | 2.3 ± 0.1 | 2.7 ± 0.1 | 2.3 ± 0.1 | <0.0001 alt |
| Umbilical venous-arterial Δ glucose (mM) | 0.72 ± 0.07 | 0.67 ± 0.09 | 0.72 ± 0.11 | 0.58 ± 0.09 | NS |
| Umbilical vein glucose delivery (mmol.min^-1^.kg^-1^ fetal weight) | 0.33 ± 0.02 | 0.25 ± 0.02 | 0.37 ± 0.02 | 0.23 ± 0.01 | <0.0001 alt |
| Fetal glucose consumption (mmol.min^-1^.kg^-1^) | 0.065 ± 0.006 | 0.056 ± 0.007 | 0.075 ± 0.008 | 0.047 ± 0.007 | <0.01 alt |
| Umbilical vein lactate (mM) | 3.35 ± 0.16 | 3.68 ± 0.13 | 3.37 ± 0.09 | 3.67 ± 0.22 | =0.06 alt |
| Umbilical artery lactate (mM) | 3.57 ± 0.15 | 3.62 ± 0.11 | 2.76 ± 0.11 | 3.96 ± 0.28 | <0.005 alt, <0.005 inter |
| Umbilical vein Insulin (pmol.ml^-1^) | 93.1 ± 14.6 | 75.7 ± 16 | 157.7 ± 28.5 | 61.1 ± 8.3 | <0.01 alt (nonparam  M-W U) |
